# Nursing staff experiences of participating in digital therapeutic product development: a qualitative study

**DOI:** 10.3389/fpubh.2026.1783292

**Published:** 2026-08-04

**Authors:** Liu Yang, Yan-Xia Han, Ye-Qiu Ye, Qian Zhao, Zhen-Yun Wu

**Affiliations:** 1 Department of Respiratory Medicine, The First Affiliated Hospital of Soochow University, Suzhou, Jiangsu, China; 2 Department of Nursing, The First Affiliated Hospital of Soochow University, Suzhou, Jiangsu, China

**Keywords:** barriers, digital health, digital therapeutics (DTx), nursing staff, qualitative interviews

## Abstract

**Background:**

Digital therapeutics (DTx) have emerged as practical tools for optimizing disease management. Due to their clinical expertise, nurses are increasingly involved in the research and development (R&D) of these products. However, while the existing literature has primarily focused on the clinical efficacy of DTx, the lived experiences and specific challenges faced by nursing staff during the development process remain largely unexplored.

**Methods:**

This qualitative study used purposive sampling to recruit 10 nursing staff members who participated in the full R&D lifecycle of DTx products. Data were collected through individual in-depth interviews and analyzed using conventional content analysis.

**Results:**

The qualitative analysis identified three major themes: (1) difficulties and challenges; (2) coping and professional transformations; and (3) needs and development. The first theme comprised subthemes including the existence of knowledge barriers, financial constraints, and challenges in team formation. The second theme included the lowering of standards, self-improvement, and seeking external assistance. The third theme addressed the cultivation of cross-specialty talent, the provision of policy support, and a focus on economic sustainability.

**Conclusion:**

Nurses’ participation in DTx product development faces multiple challenges, highlighting the need to strengthen the cultivation of cross-specialty talent and enhance policy guidance. Future efforts should focus on integrating digital technologies with healthcare to promote the high-quality development of digital health applications.

## Introduction

With the rapid advancement of information technology (IT), society has entered the digital era, and the integration of digital and healthcare technologies has become an inevitable trend. The Global Strategy for Digital Health (2020–2025), issued by the World Health Organization (WHO) in 2019, prioritized digital health strategies and recommended accelerating the digitization, networking, and intelligent construction of the healthcare field to meet new challenges and opportunities in global healthcare (1). As an emerging technology at the intersection of medicine and digital science, digital therapeutics (DTx) have gradually penetrated many aspects of healthcare, including chronic disease management, lifestyle modification, and sleep disorders (2–5).

The DTx concept first appeared in a 2007 patent. In 2017, industry pioneers established a nonprofit industry association in the United States and formulated the first definition of DTx: a software-driven, evidence-based intervention to treat, manage, or prevent disease (6). Digital therapy can be used alone or in conjunction with drugs, medical devices, or other therapies. This is accomplished primarily through information (e.g., text, pictures, and videos on an application), physical factors (e.g., sound, light, electric currents, magnetic fields, and combinations thereof), and other factors. These collectively exert a comprehensive effect on the patient to achieve optimal care and health outcomes.

Rising rates of chronic and mental illness and the ascent of “value-based medicine” continue to drive the development of DTx. Recently, DTx products based on applications or artificial intelligence (AI) platforms have been combined with wearable and/or medical devices to prevent, detect, control, or treat a variety of conditions, including psychological, cognitive, metabolic, and respiratory diseases. The popularity of the global DTx field has attracted many scholars. Unlike the United States, which is the world’s most active region in terms of DTx research and development (R&D), China’s work in this field has just begun, and its DTx products focus primarily on patient disease management.

Nursing staff are the guardians of patient disease management. Their work encompasses disease care, prevention, rehabilitation, health promotion, and other management and care layers. Nurses regularly assess patients’ chronic disease management needs and conduct timely supervision and coordinated education so that such management extends from the hospital to the home. This improves access to healthcare services and delays disease progression. Additionally, digitalization-driven chronic disease management allows nursing staff to actively explore new models of digital management, participate in R&D, and gradually enrich various DTx products, ranging from simple health education information to virtual reality (VR) technologies (7). The integration of nursing and technology is rapidly progressing. Mallik et al. (8) used interactive, immersive VR scenes to simulate real-life emergencies caused by diabetes. Patients deal with an emergency in a virtual environment, and this enhances their confidence in managing the disease while the technology collects personalized feedback and indicators from them. Propeller Health’s digital treatment for asthma and chronic obstructive pulmonary disease (COPD) improves patient adherence by pairing inhaler sensors with a smartphone application to monitor the medication duration and frequency and provide personalized support. This approach has been shown to significantly reduce asthma-related hospitalizations and emergency room (ER) visits (9). The “XiaoBeiKe” project, led by Yin et al. (10), integrates trial reports and antiviral treatment data to provide high-risk hepatitis B patients with real-time dynamic reminders, data-driven clinical recommendations, digital health education, and intervention management, with the goal of reducing mother-to-child transmission of hepatitis B disease.

Similar studies have yielded positive results for clinical efficacy and patient adherence, but their focus has centered on patient user experiences of digital therapeutics or evaluations of the technology’s effectiveness in practice (11, 12). These studies have paid little attention to the genuine feedback and perceptions of frontline clinical practitioners. In particular, there has been a lack of systematic examinations of the experiences of nursing staff, a key stakeholder group. Doctors make intermittent clinical decisions based on periodic summary data, whereas nurses are responsible for continuous bedside monitoring and must compare and verify algorithmic alerts against patients’ dynamically changing clinical conditions in real time. When the logical architecture of digital therapeutics is incompatible with nurses’ clinical reasoning, this can lead to alert fatigue or the overlooking of critical clinical signs (13). Such risks are difficult to discern from patients’ subjective experiences and are unlikely to be fully identified during doctors’ intermittent ward rounds. Additionally, the integration of digital therapeutics can increase the operational workload of nursing staff (14). This will further affect their already highly fragmented work time. Research has indicated that frequent task switching and workflow interruptions are the norm in nursing practice (15, 16), and redundant documentation, ineffective alerts, and system-enforced operations introduced by digital tools further exacerbate nurses’ cognitive loads (17, 18). If this perspective is overlooked, nurses are more likely to develop “workarounds,” bypassing the system to maintain their existing work efficiency. This behavior can fundamentally undermine the effective implementation and intended therapeutic benefits of digital therapeutics. However, less is known about nursing staff’s experiences of participating in digital therapeutic product development as active co-designers.

This exploratory qualitative study systematically examines the authentic experiences of nurses involved in the R&D and application of digital therapeutic products. It thoroughly explores the potential barriers and facilitators that influence their adoption and use in frontline clinical practice. By focusing on a stakeholder group that has been largely overlooked in digital health research, we seek to generate empirical insights into the nature of nursing participation in DTx development, the structural barriers they encounter, and the strategies they develop to sustain such engagement. Therefore, this study aims to explore the experiences, barriers, and coping strategies of nursing staff engaged in DTx development through qualitative interviews. The findings are expected to enhance understanding of nurses’ current participation status in this emerging field and to inform the development of targeted strategies to better support their active and meaningful engagement.

## Methods

### Study design

This qualitative study employed a descriptive qualitative research design. Individual, in-depth interviews were conducted to obtain detailed descriptions of participants’ experiences. The primary aim was to explore nursing staff experiences of participating in the development of digital therapeutics (DTx). The study is reported in accordance with the Consolidated Criteria for Reporting Qualitative Research (COREQ) guidelines.

### Participants

Purposive sampling was conducted to select and interview nursing staff who participated in the R&D of DTx products at four hospitals in Suzhou. Following the principle of maximum variation sampling, we purposively recruited participants from multiple DTx development teams who performed a broad range of roles within the R&D process. The inclusion criteria for this study were as follows: (1) possession of a valid registered nurse professional qualification certificate; (2) participation during any part of the product technology R&D lifecycle, including design conceptualization, clinical scenario construction, protocol development, and interdisciplinary coordination; (3) articulateness and the ability to truly express personal experiences; and (4) voluntary participation in and cooperation with this study. Participants who withdrew midway were excluded. It should be noted that, since the inclusion criteria covered participation at any stage of the lifecycle, the extent and nature of nurses’ involvement varied considerably across different projects, development phases and individual roles. Ultimately, we interviewed 10 nursing staff members. The sample size was determined based on information saturation. Following the criteria suggested by Guest et al. (19), the research team reviewed transcripts iteratively throughout the data collection period and determined that data saturation had been provisionally reached when no new codes or themes emerged from the initial interviews. To further confirm the stability of this saturation, two additional interviews were conducted. As these final interviews also yielded no new codes, the saturation was considered confirmed. The interviewee general characteristics are shown in Table 1. This study was approved by the Ethics Committee of the First Affiliated Hospital of Soochow University (approval no. 2022-236).

**Table 1:** Interviewee characteristics (*n* = 10).

Participant ID code	Gender	Age (years)	Education	Type of R&D
N1	Female	31	Master’s degree	Serious game for medication guidance
N2	Female	29	Master’s degree	WeChat mini-program for home workouts
N3	Female	37	Master’s degree	VR-based exercise intervention platform
N4	Female	30	Master’s degree	Serious game for medication guidance
N5	Female	24	Master’s degree in progress	Application for immunotherapy symptom management
N6	Female	38	Master’s degree	Online platform for health education
N7	Female	38	Master’s degree	WeChat mini-program for intestinal preparation
N8	Female	26	Master’s degree	VR-based exercise intervention platform
N9	Female	28	Master’s degree in progress	WeChat mini-program for dietary assessment
N10	Female	29	Master’s degree	Convolutional neural network evaluation system

### Procedure

This study employed a semi-structured interview approach to explore nursing staff authentic experiences during the R&D of DTx products. Based on our research objectives, we reviewed the relevant literature and consulted experts to develop an initial interview outline. To ensure the scientific validity of this outline, pre-interviews were conducted with two nurses prior to the formal interviews. The outline was refined based on the results of these pre-interviews. The final interview outline included the following questions (Table 2).

**Table 2:** Interview guide.

No.	Interview outline
1	Please talk about your experience with the R&D of DTx products.
2	What are the reasons for your involvement in the R&D of DTx products?
3	What are your thoughts after participating in the R&D process?
4	What difficulties have you encountered while participating in DTx R&D, and how did you deal with them? What kind of help do you think you need during this process?
5	How do you perceive the future evolution of digital therapeutic products, and do you have any ideas or suggestions?

The interviews were conducted by the first author in a hospital conference room. Prior to each interview, the interviewer established rapport and built trust with the participant, explaining the purpose and significance of the study. The participants were encouraged to share their experiences and provide additional comments. Interviews were audio-recorded after obtaining written informed consent from all participants. Participants were assured of the confidentiality of their personal information. The duration of each interview was 30–40 min. The interviews were conducted in a quiet, comfortable room within the hospital selected specifically for this purpose. The researchers observed and recorded all linguistic and non-linguistic data—including facial expressions, tone of voice, and body language—exhibited by the participants. These observations were documented in field notes and used to contextualize the verbal responses during data analysis. The audio recordings were transcribed verbatim, and the field notes were organized within 24 h of each interview. The transcripts were then rechecked, organized, and stored on an institutionally approved, password-protected, encrypted cloud storage platform that was accessible only to the research team. All data will be retained for five years following study completion prior to permanent deletion. The interview transcripts were also anonymized to protect participant confidentiality.

### Data analysis

Inductive content analysis was used to analyze the data following these steps (20): (1) the interview transcripts were read repeatedly to achieve a comprehensive understanding of the data, and key ideas and perspectives were annotated; (2) using the sentence as the unit of analysis, the transcripts were openly coded; and (3) similar and related codes were grouped into themes and subthemes, and the thematic structure was refined based on internal connections and logical coherence, with representative quotations selected. To enhance analytical rigor, two researchers (YXH, YQY) independently coded all transcripts, compared their coding structures, and discussed any discrepancies until consensus was reached through team deliberation.

### Methodological rigor

To enhance representativeness among the nursing staff and strengthen the credibility of the findings, participants were selected to reflect variations in their R&D roles, the types of digital therapeutic products involved, and other relevant characteristics. All researchers who conducted the interviews had completed formal training in qualitative research methods. In addition, no teaching or supervisory relationships existed between the researchers and participants, thereby minimizing the potential for power imbalances or response bias. Prior to the formal interviews, the researchers established rapport with participants, refined their interview skills, and conducted pilot interviews. During the interviews, they adopted active listening skills and sought to suspend any existing assumptions. Recognizing that all researchers were registered nurses with clinical backgrounds—a position offering both insider access and the potential for preconceptions—we proactively implemented reflexive strategies throughout the study. Reflexivity was maintained throughout the entire research process, including the stages of developing the interview guide, data collection, coding, and theme construction. Structured reflexive journals were used to document the researchers’ preconceptions prior to the interviews, reflections on communication styles and probing strategies immediately after the interviews, and thoughts on how their positionality may have influenced coding and interpretation. These research records were reviewed during team debriefing sessions. When risks such as the confirmation bias were identified, procedural adjustments were made, and discrepant cases were re-examined.

## Results

**Figure 1:**
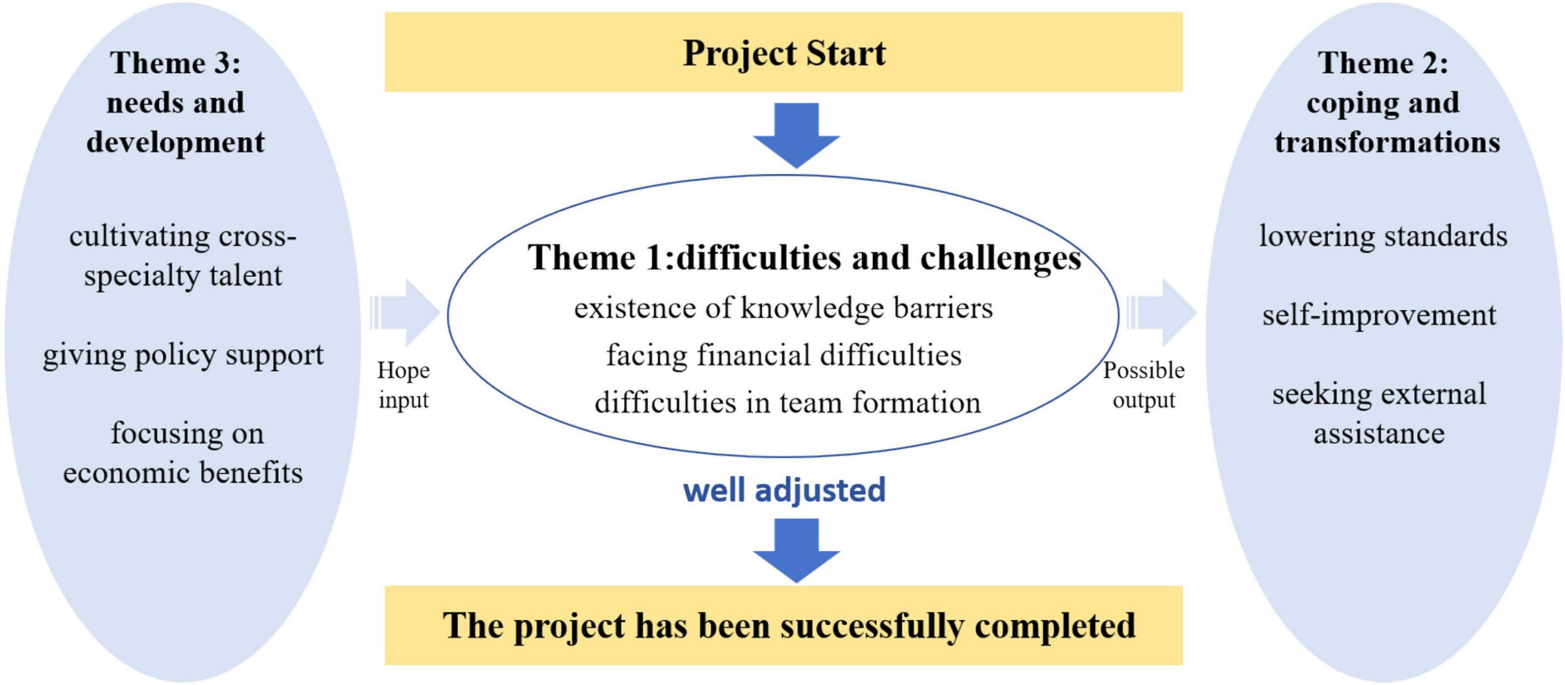
Participant experiences in the development of digital therapeutics. During the early stages of digital therapeutic development, nursing staff faced a variety of difficulties and challenges. In response, they were required to cope and transform to maintain forward project momentum. Throughout this process, they gradually recognized more profound systemic needs and development areas that included the cultivation of cross-specialty talent, the need for policy support, and a focus on economic benefits. The participants considered these conditions essential to move projects toward success.

Three themes comprising eight subthemes were identified in this study: (a) Difficulties and challenges, which included the existence of knowledge barriers, financial difficulties, and team formation difficulties; (b) Coping and transformation, which included the lowering of standards, the pursuit of self-improvement, and actively seeking external assistance; and (c) Needs and development, which addressed the cultivation of cross-specialty talent, the need for policy support, and a focus on economic benefits. Figure 1 and Table 3 present the themes, subthemes, and representative quotes.

**Table 3:** Themes and subthemes of the study.

Themes and subthemes	Number of interviews (*n* = 10)	Representative quotes
Difficulties and challenges		
Existence of knowledge barriers	6	“I do not really understand the technical stuff.” “I cannot tell whether what they said is right or not.” “The development pace was completely controlled by the other side.”
Facing financial difficulties	10	“The quotation was much higher than we expected.” “In the end, we had to cover the costs ourselves.” “We’ve received no funding.”
Difficulties in team formation	10	“I did not know where to find the right people to do this.” “The team we found at the beginning wasn’t a good fit.”
Coping and transformations		
Lowering standards	6	“We could not meet our expectations, but there was no solution.” “Some problems just could not be solved, so we had to give up.”
Self-improvement	5	“I learned a lot of new things.”
Seeking external assistance	7	“A classmate gave me some help.” “I actively sought help.”
Needs and development		
Cultivating cross-specialty talent	9	“Having interdisciplinary colleagues around me was very helpful.” “We need more knowledge translators.”
Giving policy support	7	“We hope there will be an institution to provide guidance.” “We need financial support.”
Focusing on economic benefits	6	*“*We cannot charge for this yet.” “We’re not receiving any revenue.”

### Theme 1: difficulties and challenges

#### Existence of knowledge barriers

The development and application of DTx products require not only medical knowledge but also programming, art, design, operation, and other technical skills. All participants acknowledged that their limited interdisciplinary knowledge constrained their ability to effectively oversee product development. This barrier manifested in three interrelated manners: difficulties in understanding the technical terminology and evaluating feasibility, challenges in drafting and negotiating contracts, and a perceived loss of control over the R&D process. N4 described the communication gap she encountered when discussing technical specifications with developers: *“When I communicate with the people at the game development company, I do not know much about the things they describe, such as the choice of H5 game, APK form, or domain name. They perceive my expectations as excessively high…. Consequently, I find it challenging to discern the veracity of their statements.”* N9 faced similar difficulties during the contractual phase: *“I had absolutely no idea what to look for when signing a contract.* […] *Later, when I asked the other party to make a request, they said that this (content) was not in the contract. The contract instead became a regulation that bound me”.*

#### Financial difficulties

The development of DTx products requires varying levels of financial support, and a majority of the participants indicated that they had experienced funding challenges that included self-funding due to a lack of external support, unexpected post-development costs, and the perceived inflation of initial quotations. Several participants described paying R&D costs out of their own pockets or using their supervisors’ research funds, a practice they acknowledged was unsustainable. N1 explained the financial burden of her postgraduate project: *“I must cover the costs myself. I have thought about other methods to raise funds but have not been able to achieve them. In the end, I paid for the cost myself. Of course, this method is not desirable.”* N5 similarly described compromises made due to limited funding: *“To reduce costs, sometimes even when there were new methods that were more suitable for the platform’s functional design, the financial constraints forced me to just settle for what we had”*.

Others faced unexpected maintenance and operational costs after the initial development phase.

N7 noted: *“When I was developing it, I thought it would be done once the development was finished. But later they said there would be additional charges for maintenance and operation…I directly posted it myself, but I do not know how long this enthusiasm can last”*.

Participants also expressed difficulties in judging whether commercial quotations were reasonable.

N3 reflected on her experience: *“Our project was supported by research funding and cost over CN¥200,000. From an IT perspective, I’m not very satisfied with the result. I think the cost was too high…* [sigh] *I still became the ‘injustice’ [victim of unfair pricing]”*.

#### Difficulties in team formation

The participants reported that forming an R&D team was difficult and that teamwork was often problematic, particularly regarding cooperation and communication with information engineering teams. These challenges elicited varying degrees of negative emotions. N2 struggled to find local partners: *“I wanted to find a local team to work with, but I have not been able to find one. Online communication sometimes wasn’t so smooth.”* N1 described her difficulty navigating the digital industry’s specialized divisions: *“It was only after I got involved in development that I realized network companies have specialized areas… Those capable of developing public accounts and apps may not necessarily be able to develop games. Some might even be intermediaries… But I did not know where else to find people to do it”.*

Negative collaboration experiences were common.

N8 expressed frustration with a partner that repeatedly resisted improvement requests: *“During our later collaboration, we found that every time we suggested an improvement, they either asked for more money or claimed it could not be done* [said excitedly]*. This greatly affected our willingness to redevelop”*.

### Theme 2: coping and transformation

#### Lowering standards

When faced with insurmountable difficulties, six participants reported lowering their initial expectations for their products. This was not a preferred strategy but a pragmatic response to constraints.

N1 described the gap between her expectations and the actual outcomes: *“Every time the company delivered a sample, I felt it did not meet my expectations, but I could not provide them with specific standards… But there was nothing I could do to change it.”* N3 similarly expressed dissatisfaction with the standard of the work delivered: *“The team obviously had designers and planners, but it seemed like they did not exist. They ignored the professional content… It felt like a makeshift team with no professionalism!”* For N7, the compromise was more granular: *“I wanted to make the fonts on some of the app’s interfaces more eye-catching and vivid…But they could not do it, so I eventually gave up”*.

#### Self-improvement

All participants expressed a willingness to acquire new skills to compensate for the knowledge gaps they encountered, though they acknowledged the limitations of this strategy. N5 explained the motivation to upskill: *“DTx technology is the inevitable product of the information society. If you want to adapt to the needs of social development, you need to continue to learn and improve yourself. For example, to figure out R&D costs, I have specifically studied certification fees, domain names, servers, and the front- and back-end development costs of APPs.”* N4 acquired video production skills through self-study: *“I taught myself video shooting and editing, and now I can proficiently use several editing applications* [laughter]*”*.

However, self-improvement had its limits.

N1 described dubbing an application herself to avoid additional costs: *“I did not realize that the sound effects would pay extra money* [frown]*, so I dubbed it myself after learning* [sigh]*. But after all, I’m not a professional* [voice actor]*, and the final rendering is still weird.”* N8 expressed frustration at having to solve problems that should have been addressed by the collaboration team: *“While developing a digital platform, I really learned a lot from it, but I always had to solve everything myself… This was clearly work that the collaborative R&D team should have been doing* [said angrily]*!”*.

#### Seeking external assistance

Five participants actively sought help from their personal networks. N1 consulted a lawyer friend for a contract review: *“I had never signed a contract before. When I needed to sign one for product development cooperation, I was afraid I did not understand it. I asked my lawyer friend to help me review it, and we did find some issues.”* N8 sought guidance from her sister, who had prior experience with similar projects: *“I asked my older sister for guidance on the user manual… because she had developed a similar* [manual]*.”* N10 relied on peers: *“Luckily my classmates helped me solve a lot of difficulties; otherwise, I would not have known what to do”*.

However, two participants described unsuccessful attempts to seek external help. N3 sought expertise on rehabilitation devices: *“I wanted to find someone knowledgeable about the Internet of Things to consult. But it seemed that everyone is quite vague about it, and I could not find anyone more informed.”* N10 similarly struggled to find expert support for her technical queries: *“I want to have an in-depth understanding of convolutional neural networks, and I want to find someone to help me with my ideas, but I have not been able to. Perhaps it’s because the resources available to me are limited, and no one can help* [said with a bitter smile]*”*.

### Theme 3: needs and development

#### Cultivating cross-specialty talent

The vast majority of participants identified the cultivation of cross-specialty talent as a critical need. N2 articulated the role of such individuals: *“The R&D team for DTx product technology is divided into two major sections: digital and medical. If we want to communicate without barriers, we need a translator who understands both the medical and the digital sides. However, I currently do not have such a translator around, so I have to do it myself.”* N4 reflected on the communication challenges she encountered with engineers: *“When I was communicating with the engineers, I found that they did not understand the inhalation medication in my program at all… I felt that communication was just too difficult”*.

N8 provided a concrete example of the value of cross-specialty talent: *“I have a college classmate who majored in nursing for her undergraduate degree but switched to a computer-related field for her graduate studies. When I explained my needs to her, she understood immediately and told me how to communicate them to others. After I followed her advice, it was indeed much more efficient and smoother”*.

One participant thought that cross-specialty talent might not always be necessary for R&D: *“The functionality of my program is relatively simple… but perhaps for more-complex projects, cross-functional talents might be required”*.

#### Providing policy support

Participants identified policy support, including institutional guidance, dedicated funding mechanisms, and reimbursement frameworks, as a necessity for sustainable DTx development. N1 argued for government-led coordination: *“I think that if a governmental unit or administrative department, such as the Health Commission, takes the lead to provide long-term cooperation as well as specialized medical and health-related information… this will be more economically and socially cost-effective than seeking a partner company ourselves”*.

N8 similarly advocated for institutional support: *“We could collaborate with computer science students from universities for joint development… or the community could offer similar public-service guidance (government-led)… But it feels like people aren’t paying much attention to this now, and I have not seen any relevant policy support”*.

N9 highlighted the financial dimension: *“Cost is a major issue. Generally, there is funding support for R&D, but the later application and maintenance of the results often require money… It would be great if there were relevant policy assistance; then I would not have to worry so much and could fully focus on getting the job done”*.

#### Focusing on economic benefits

Six participants emphasized the importance of considering economic benefits, noting that the absence of revenue mechanisms undermined the sustainability of DTx projects. N1 identified this as a systemic issue: *“The inability to generate revenue, and even the need for healthcare professionals to cover additional expenses, may be important factors that hinder the development of many DTx product applications.”* N3 planned to obtain new technology certification to enable reimbursement: *“It has been proven that our commissioning technology can change patient outcomes. I intend to apply for a new technology certification to implement it as a clinical-treatment option, which will benefit patients while also generating economic returns, ensuring the sustainable application of this technology in the future”*.

N4 observed that neglecting commercial value limited the quality of their products: *“In terms of user experience alone, our product lags significantly behind apps available in mobile-app stores. Probably because we only use the product as a technology for R&D, ignoring the commercial value it could bring…”* N9 similarly argued for a balanced approach: *“We should prioritize meeting patients’ true needs while integrating economic benefits with social benefits to ensure the long-term feasibility of patient disease management”*.

## Discussion

### The cultivation of cross-specialty talent and the formation of interdisciplinary teams

The R&D of DTx products involves both digital and medical dimensions; hence, cross-specialty cooperation is necessary. In this study, we identified several challenges in the technology integration process, particularly communication and collaboration barriers between partner organizations arising from knowledge gaps, which contributed to suboptimal R&D outcomes. For example, in the development of nursing-related functional games (21, 22), companies serve as technology providers responsible for project development, with team members specializing primarily in computer science, software engineering, marketing, animation, and art design. Given the professional knowledge thresholds of both parties, cross-disciplinary and multi-skilled professionals who can serve as “translators” are crucial for team formation. However, individuals with such backgrounds are in short supply, accounting for only 4.9% of team members (23), and they are primarily concentrated at large internet companies like Tencent and Shanda. This shortage of interdisciplinary talent has led to a notable phenomenon where, in the current environment, nursing staff are often required to assume a leading role in DTx product development, taking on substantial responsibilities in research and design. This is not because they are the most qualified, but because no other personnel can bridge the gap between clinical practice and technology (24). The construction of this “default leadership” model is also determined by the structural characteristics of China’s current digital therapeutics (DTx) market. We found that, due to limited digital technology knowledge (25), nurses involved in digital R&D projects tend to focus on developing health management platforms such as web-based systems, applications, mobile applications, and mini-programs. These are areas in which clinical expertise is essential and technical complexity is relatively manageable. However, as participants repeatedly emphasized, internet companies have limited willingness to independently develop such products, primarily because clinical promotion is unlikely to yield clear economic benefits (26).

Therefore, as commercial entities withdraw while clinical needs persist, nursing staff are likely to continue playing a central role in DTx development in the foreseeable future, not as an optimal arrangement, but as a response to a lack of interdisciplinary collaboration. However, this may also position nurses as potential incubators of cross-disciplinary talent, as sustained involvement in R&D enables them to acquire both clinical and technical skills through experiential learning.

The importance of cultivating such cross-specialty talent is increasingly recognized internationally. To support the development of digitally competent nursing professionals, the European Union launched the “2021–2027 Digital Education Action Plan,” which requires member states to strengthen digital infrastructure and education systems while enhancing the digital skills and literacy of students across countries (27). Under this framework, the Department of Nursing at the University of Turku in Finland has established a Future Health Technology program, integrating digital information courses such as VR, augmented reality, and application design into its health technology module (28). In China, the School of Nursing at Fudan University (Shanghai, China) launched an interdisciplinary curriculum for graduate nursing education in 2019 that is also open to students from related research areas in the School of Nursing and the School of Information and Engineering (29). The curriculum includes courses on body and health assessments, as well as intelligent medical systems that integrate neural sensing, VR, mixed reality, digital health technologies, and global healthcare ethics. It has received positive evaluations since its implementation. These international and domestic initiatives are consistent with our empirical observation that the demand for cross-specialty talent is a systemic challenge that requires structural solutions, and that nursing staff, given their sustained frontline engagement, are uniquely positioned to benefit from such educational interventions. Consistent with our finding regarding knowledge-driven communication barriers in nurse-led DTx projects, it is recommended that hospital administrators actively recruit such professionals and establish dedicated teams, or provide continuing education in general or digital literacy for nursing staff through seminars, lectures, and online courses (30). By keeping curricula up to date with topics such as big data, cloud computing, artificial intelligence (AI), and smart hospitals, healthcare institutions can further cultivate interdisciplinary digital talent and promote the high-quality development of digital health technologies. Such institutional efforts are particularly important in the Chinese context, where external technical support remains limited and nursing staff continue to fill critical R&D roles. More fundamentally, while interprofessional collaboration is widely recognized as a cornerstone of digital health development, our findings revealed a specific configuration of such collaboration that has received limited attention in the existing literature: one in which nurses assume leadership not by design but by default, shaped by the scarcity of cross-specialty translators and the withdrawal of commercial entities in an immature market. This observation extends the current understanding of interprofessional dynamics by demonstrating that the form of collaboration is not uniform across contexts, but it is profoundly conditioned by local institutional and market structures. These findings indicate that such nurses’ leadership roles in DTx development are context-specific, individually shaped by organizational readiness, institutional support for digital innovation, the maturity of local health informatics infrastructure, and the specific clinical domain of the DTx product. With the ongoing professionalization of digital nursing in China, these leadership roles are increasingly coexisting with emerging professional roles in nursing informatics and digital health.

### Clarifying the direction of the digital nursing industry and enhancing the vitality of new technological strategies

In this study, we found that DTx product development projects involving nursing staff are fragmented and diverse, with limited overall coordination, and generally operate in isolation, which hinders patients from benefiting continuously from these interventions. Additionally, given the current level of technical development, these projects tend to have relatively low technical requirements and are often focused on single thematic areas. Although they adopt various formats (e.g., applications, the Internet of Things [IoT], games, and VR), they often lack effective design for user engagement and exploratory interactions. To some extent, this reflects the current direction of domestic nursing-led exploration in digital nursing technologies. Given these observed limitations in coordination and user interactivity, achieving a fundamental breakthrough necessitates a strategic shift from isolated, project-based initiatives toward a more cohesively integrated approach. This approach will recenter on the foundational principles of nursing practice to critically reexamine the core mission and essential characteristics of DTx products. DTx refer to software-driven, evidence-based intervention programs designed to treat, manage, or prevent disease. As an essential component of healthcare delivery, nursing encompasses the continuous monitoring and support of patients’ physiological, psychological, and social adaptation throughout the entire care process. With the rapid advancement and continuous innovation of digital technologies, the development of digital hospitals, intelligent nursing, and related innovations has become a powerful trend. Within this evolving environment, nurses must clearly understand the direction of digital transformation in traditional nursing, adapt accordingly, and actively contribute to the high-quality development of the profession.

In light of this, to address the fragmented and isolated nature of current projects, it is first necessary to consider the aspects of nursing that may generate transformative “butterfly effects.” Nursing serves all individuals with healthcare needs, and its scope has expanded beyond disease care to encompass the entire spectrum from health to illness, including disease prevention, management, and treatment across populations. Furthermore, nursing services are no longer confined to hospital settings. For example, the establishment of internet-based nursing platforms not only broadens the scope of services but also facilitates the integration of medical resources and breaks down the data silos between individual DTx products. Such platform-based integration provides a clear way to overcome the project-by-project isolation we observed, enabling continuity in patient health management, and better meets patients’ diverse needs. Zhejiang’s online “Zheli Nursing” service bridges the “last mile” of medical services while innovating the frontier of nursing practice (31).

In addition, considering the relatively low technical requirements, nursing practice has expanded to include digital care approaches such as online health information delivery, the establishment of health records for patients with chronic diseases, guidance and monitoring of patients’ home self-care, and the application of novel health rehabilitation measures (32–34). These initiatives provide comprehensive and continuous support for patient health management. While maintaining a focus on patient care, nursing education and management have also seen significant progress in this transformation. For example, real-time information exchange enables nurses to adjust care plans in response to clinical needs using personal digital assistants (PDAs), thereby enhancing the digitization and automation of nursing workflows (35). Such workflow-oriented digital tools align well with the manageable technical thresholds described by our participants, suggesting that the incremental digitisation of routine nursing processes may be a more feasible priority in the near term. The use of IoT technologies and sensors also enables real-time monitoring and detection of patients’ physiological parameters. These developments are tangible manifestations of the transition toward the digital transformation of clinical nursing quality.

VR can provide nursing students with a safer and more realistic training environment (36), and such immersive technologies could similarly be adapted to resolve the lack of exploratory interactions for patients. In addition, the digital transformation of nursing cannot be achieved without the support and drive of data elements. The circulation and application of data elements facilitate the integration of multi-source data throughout the entire health service process, including prevention, treatment, and rehabilitation. Currently, healthcare systems rely on smartphones and wearable devices to provide real-time feedback on patient health status, analyze the gaits of stroke patients during rehabilitation (37), or deploy nursing robots to assist with self-rehabilitation. Continuously emerging, widely applied advanced IT methods such as cloud computing, the internet, big data, and VR are increasingly being used in various areas of nursing work, including chronic disease management, health education, nursing quality management, and nursing education and training. Collectively, these technologies unlock the potential of digital health and further advance the digital transformation of nursing.

Furthermore, a wave of technological innovations has emerged in frontier fields. This is exemplified by generative AI tools such as ChatGPT (released by OpenAI in 2022). Their potential applications in the nursing field include disease management support, health education, patient data collection, and needs assessments, all of which could effectively enhance user engagement by providing highly interactive experiences, which warrant further investigation (38). However, the introduction of such sophisticated tools carries the risk of inadvertently widening the digital divide for the main beneficiaries of chronic disease management: the older adult population. Consequently, significant challenges persist in terms of expanding the scope of clinical applications and promoting universal access to DTx products. Addressing these challenges will require balanced, multidisciplinary, and context-sensitive approaches.

### Emphasizing policy to drive technological improvement and exploring the healthy development of the nursing information industry

Advances in digital technologies have accelerated the integration of healthcare and information technology, ushering in a new era of digital health. As the primary drivers of DTx implementation and management, nursing staff are actively exploring the application of DTx across a wide range of disease areas. As a result, the diversity of DTx products continues to expand, with inventions constantly emerging. However, the wider DTx market is still relatively fragmented, and its commercialization lacks a mature model. As an innovative integrated field, DTx product development requires collaborative efforts from research institutions, software companies, medical institutions, and other stakeholders. However, such cooperation remains fragmented, with limited scale and depth. Information enterprises lack clarity regarding R&D directions, clinical indicator settings, and other critical issues, and these must be addressed in the healthcare sector. However, high upfront investments required during early product development stages, combined with an unclear profit-sharing model for long-term benefits, deter enterprises from participating due to profitability pressures.

In this study, we found that digital R&D projects involving nursing staff are often confined to individual research topics and are highly dependent on the interests and efforts of individual investigators. Most participants expressed a need for timely policy support to help address challenges throughout the R&D process. Specifically, our data showed that nurses lacking formal training in intellectual property and contract law become highly vulnerable to contractual disputes and cost overruns with commercial vendors when forced to lead projects. This vulnerability makes a clear regulatory framework not just beneficial, but operationally imperative. Currently, the relevant approval and regulatory policies for DTx in China remain in the exploratory stage. We must urgently establish effectiveness and safety standards tailored to the rapid updates, frequent iterations, and clinical applications of DTx products to gradually improve the relevant policies and regulations that support these processes. Driven by the consensus among participants regarding the need for clearer regulatory frameworks, the government can play a leading role by establishing industry organizations, such as information committees, or by selecting and compiling directories of qualified information enterprises. To nurture cross-disciplinary medical and engineering talent, institutions can provide retraining for in-service personnel in related fields to encourage cross-industry development (39). This approach would help the nursing staff who lead DTx R&D efforts address contract loopholes, inflated R&D expenses, and other challenges, thereby achieving their expected R&D goals (40).

Given that current DTx development is predominantly undertaken by nursing staff who often lack systematic training in software engineering and cybersecurity, R&D projects are generally confined to low-complexity applications and commonly proceed without dedicated technical oversight from IT security professionals during the design phase. Since DTx are software-driven products that necessarily require access to wide-area networks (WANs) during their application and promotion, this absence of technical oversight may directly amplify risks such as the leakage of sensitive personal information and unauthorized remote control (41). Based on product characteristics, R&D and production units should explore methods for the monitoring and early warning of data security risks while establishing and improving a digital security guarantee system for DTx products that covers their entire lifecycle. To ensure the safety and compliance of digital medical products, governmental entities must establish a collaborative consultation framework and an inclusive regulatory mechanism that improves the relevant legislation and implements categorized supervision of digital medical products, such as applications, mini-programs, or websites, based on the potential risk of harm or type of medical service.

We also found that cost support should not be ignored in the R&D and promotion of DTx products. A majority of the participants stated that the lack of financial support seriously hindered the continuous operation of their programs. DTx products developed with the help of nursing staff are primarily interventions based on online software or intelligent hardware that primarily focus on the management of chronic-disease health behaviors (42). Their public-welfare value is significant, but their commercial attributes are weaker. Therefore, hospitals should be encouraged to cooperate on DTx innovation pilot projects and establish reasonable incentive mechanisms and special funds to support the R&D, application, and upgrading of DTx products. Hospitals should also develop coherent services ranging from early intervention to the effective diagnosis and treatment of diseases through these products. This would assist in creating a multi-layered service system that includes rapid screening, remote initial diagnosis, in-hospital diagnosis, and DTx interventions for rehabilitation, providing patients with full-cycle, disease-specific management. Additionally, hospitals could establish dedicated nursing informatics positions to complement, rather than replace, the informal leadership roles identified in our study. These formalized roles would anchor nursing expertise within the digital health ecosystem, enabling the continuous monitoring of patients’ health behaviors and lifestyles to ultimately improve health outcomes and alleviate the disease burden in key areas (43).

Nevertheless, the sustainable development of digital nursing cannot solely rely on policy support. The successful integration of DTx into the healthcare market is equally essential. In this study, we found that although the clinical value of DTx products was widely recognized, their usability and user engagement remained suboptimal, resulting in variable levels of patient acceptance and trust. This finding suggests that researchers should conduct in-depth market research prior to developing digital technologies to fully understand the needs and wishes of consumers and the size of the target group. This will strengthen stakeholder cooperation and facilitate a comprehensive understanding of the factors that contribute to DTx acceptance and adoption. From the user’s perspective, the incorporation of representatives of the target population into the R&D team can enhance product adaptability, avoiding the dilemma of a product being “useful but not used,” thus supporting product application and promotion.

### Strengths and limitations of this study

The first limitation of this exploratory qualitative study was that a relatively small sample of participants was recruited. However, data saturation was determined to have been achieved following the criteria suggested by Guest et al. A second limitation is that all participants in this study originated from the same province, meaning that their experiences were influenced by regional, economic, social, and other environmental similarities. Therefore, we recommend conducting similar studies in other regions to gain a more comprehensive understanding of nurses’ experiences during the development of DTx products. Third, although we did not impose any educational restrictions during recruitment, our participants were predominantly nurses with Master’s degrees working in tertiary hospital settings. While this profile reflects the population most likely to assume leadership and innovation-oriented roles in DTx R&D under the current Chinese context, given the field’s nascent stage and the limited digital health training available to the broader nursing workforce, it nonetheless limits the transferability of our findings to nurses with different educational backgrounds (e.g., diploma levels) or those working in community and primary care settings. Future research should include a more diverse range of nursing profiles to examine how participation patterns and perceptions may vary across different educational and clinical contexts.

This study contributes to the literature on nursing and digital health innovations by highlighting a stakeholder group that has received limited attention in existing research: nursing staff who assume multifaceted roles in DTx R&D. Previous studies have largely focused on patient experiences or technological effectiveness. In contrast, we have provided empirical evidence regarding the specific challenges (e.g., knowledge barriers, funding shortages, and team formation challenges) and coping strategies of these nurse innovators. Crucially, we revealed that nurses assume leadership not by design but as a default response to structural constraints, such as the scarcity of cross-specialty translators and the withdrawal of commercial entities from an immature market. Our findings offer actionable insights for policy and educational initiatives aimed at sustaining nursing-led digital health innovation in emerging markets.

## Conclusion

Through in-depth qualitative interviews, this study explored the authentic experiences of nursing staff participating in the digital healthcare R&D process. This exploration captured their challenges, the coping strategies they adopted, and their evolving needs and perspectives on future development. Based on these findings, we examined the necessity and urgency of cultivating cross-specialty nursing talent and building multidisciplinary teams for DTx development. As the nursing profession undergoes digital transformation, it must adapt to emerging trends and evolving healthcare needs by identifying innovation opportunities, strengthening digital competencies, and integrating information technologies into nursing practice. In addition, the respective roles of the government and the market in the R&D and application of DTx products should be fully recognized. Supportive policies, effective regulatory frameworks, and appropriate market incentives are essential to facilitate the standardized, sustainable, and high-quality development of digital nursing.

## Data Availability

The original contributions presented in the study are included in the article/supplementary material, further inquiries can be directed to the corresponding authors.

## References

[1] World Health Organization. Global Strategy on Digital Health 2020–2027. Geneva: World Health Organization (2025).

[2] Des Roches CA Balachandran I Ascenso EM Tripodis Y Kiran S. Effectiveness of an impairment-based individualized rehabilitation program using an iPad-based software platform. Front Hum Neurosci. (2015) 8:20150105. doi: 10.3389/fnhum.2014.01015, 25601831 PMC4283612

[3] Berman M Guthrie N Edwards K Appelbaum K Njike V Eisenberg D . Change in Glycemic control with use of a digital therapeutic in adults with type 2 diabetes: cohort study. JMIR Diab. (2018) 3:e4. doi: 10.2196/diabetes.9591, 30291074 PMC6238888

[4] Taylor DJ Peterson AL Pruiksma KE Young-McCaughan S Nicholson K Mintz J. Internet and in-person cognitive Behavioral therapy for insomnia in military personnel: a randomized clinical trial. Sleep. (2017) 40:40. doi: 10.1093/sleep/zsx075, 28472528

[5] Choi MJ Kim H Nah HW Kang DW. Digital therapeutics: emerging new therapy for neurologic deficits after stroke. J Stroke. (2019) 21:242–58. doi: 10.5853/jos.2019.01963, 31587534 PMC6780014

[6] Alliance DT. Digital Therapeutics Definition and core Principles. Available online at: https://dtxalliance.org (2024).

[7] Lu X Niu M Han Y Miao X Wu Z Zhao Q. Development of a virtual reality-based fear of Dyspnea adaptation program for patients with chronic obstructive pulmonary disease. Mil Nurs. (2023) 40:52–5. doi: 10.3969/j.issn.2097-1826.2023.09.013

[8] Mallik R Patel M Atkinson B Kar P. Exploring the role of virtual reality to support clinical diabetes training-a pilot study. J Diabetes Sci Technol. (2022) 16:844–51. doi: 10.1177/19322968211027847, 34210183 PMC9264436

[9] Merchant RK Inamdar R Quade RC. Effectiveness of population health management using the propeller health asthma platform: a randomized clinical trial. J Allergy Clin Immunol Pract. (2016) 4:455–63. doi: 10.1016/j.jaip.2015.11.022, 26778246

[10] Yin X Han G Zhang H Wang M Zhang W Gao Y . A real-world prospective study of mother-to-child transmission of HBV in China using a Mobile health application (shield 01). J Clin Transl Hepatol. (2020) 8:1–8. doi: 10.14218/jcth.2019.00057, 32274339 PMC7132014

[11] Yi Z Xin Z Hongshu W Kaibo Z Shengdi L Caiqi X . Application of digital therapy in post-operative rehabilitation of knee anterior cruciate ligament reconstruction. Chin J Med Instrum. (2023) 47:487–91. doi: 10.3969/j.issn.1671-7104.2023.05.004, 37753884

[12] Qiao Y Chang H Yang X Wang R Wei N Wang J. Evaluation of the effect of long-term full-dose cognitive digital therapy on cognitive function in patients with cognitive disorders. Chin J Pract Nurs. (2024) 40:1863–70. doi: 10.3760/cma.j.cn211501-20230925-00625

[13] Erdogan H Ulcay D Afsar F. Beyond alarm fatigue: nurses' experiences of behavioral nudge fatigue in digital clinical environments. BMC Nurs. (2026) 25:20260327. doi: 10.1186/s12912-026-04592-1, 41896820 PMC13032586

[14] Walzer S Armbruster C Mahler S Farin-Glattacker E Kunze C. Factors influencing the implementation and adoption of digital nursing technologies: systematic umbrella review. J Med Internet Res. (2025) 27:e64616. doi: 10.2196/64616, 40743516 PMC12355146

[15] Liu Q Lu Y Zhai S Dai C Kan C Chen C. Workflow interruptions, perceived workload and missed nursing: their impact on nurses' health status-a structural equation model. J Adv Nurs. (2026) 82:311–20. doi: 10.1111/jan.16855, 39991959

[16] Lin T Feng X Gao Y Li X Ye L Jiang J . Nursing interruptions in emergency room in China: an observational study. J Nurs Manag. (2021) 29:2189–98. doi: 10.1111/jonm.13372, 33993569

[17] Park J Nahm ES. Clinical nurses' point-of-technology use and digital health competency: scoping review. Comput Inform Nurs. (2026) 44:e01465. doi: 10.1097/cin.0000000000001465, 41562426

[18] Asgari E Kaur J Nuredini G Balloch J Taylor AM Sebire N . Impact of electronic health record use on cognitive load and burnout among clinicians: narrative review. JMIR Med Inform. (2024) 12:e55499. doi: 10.2196/55499, 38607672 PMC11053390

[19] Guest G Bunce A Johnson L. How many interviews are enough? An experiment with data saturation and variability. Field Methods. (2006) 18:59–82. doi: 10.1177/1525822X05279903

[20] Elo S Kyngäs H. The qualitative content analysis process. J Adv Nurs. (2008) 62:107–15. doi: 10.1111/j.1365-2648.2007.04569.x, 18352969

[21] Qian Z Mei'e N Yanxia H Zhenyun W. Development and usability assessment of serious game featuring inhalant use gui-dance for COPD patients. J Nurs Sci. (2023) 38:98–102. doi: 10.3870/j.issn.1001-4152.2023.23.098

[22] Ma X Song K. Rehabilitation therapy for children with autism based on interactive VR-motion serious game intervention: a randomized-controlled trial. Front. Public Health. (2025) 13:13. doi: 10.3389/fpubh.2025.1628741, 40977792 PMC12443706

[23] CNG China Functional Game Talent Report. Available onlie at: https://mp.weixin.qq.com/s/TDrpMc1uIqom83YAfEMEcA (2019)

[24] Teo JYC Wang W. Interdisciplinary collaboration for chronic illness prevention and management through digital health interventions. Interdiscip Nurs Res. (2024) 3:199–200. doi: 10.1097/nr9.0000000000000073, 39734596 PMC11670911

[25] Dermody G Wadsworth D El Haddad M Prichard R Benson A Benson T . Bridging the digital divide: a multi-method evaluation of nursing readiness for digital health technology. J Adv Nurs. (2026) 82:3752–66. doi: 10.1111/jan.70105, 40762402 PMC12994680

[26] Fang Q Liang J Jiao X Liu Y Zhang L Zhang H . Development and trends of digital therapeutics industry in China:a multidimensional analysis based on approved products by China's National Medical Products Administration. Chin Gen Pract. (2026) 29:232–9. doi: 10.12114/j.issn.1007-9572.2025.0167

[27] European Union. Commission E Communication from the Commission to the European Parliament, the Council, the European economic and social Committee and the Commit of the Regions—Digital Education action plan 2021-2027: Resetting Education and Training for the digital age. Available online at: https://eur-lex.europa.eu/legal-content/EN/TXT/?uri=CELEX:52020DC0624 (2020).

[28] Univetsity of Turku. Master’s Degree Programme in Future Health & Technology. Available online at: https://opas.peppi.utu.fi/en/programme/87490?period=2022-2024 (2022).

[29] Ge X Jia S Hu Y Zou Z Guo X Xiang L. Exploration and practical research of cultivating postgraduate students with master of nursing specialist in the field of health informatics. Chin J Nurs Educ. (2024) 7:828–34. doi: 10.3761/j.issn.1672-9234.2024.07.010

[30] Yueliang Z Xinyi L Shixi H. Practice and enlightenment of American public libraries' digital literacy education. Doc Inform Knowl. (2021) 38:21–37. doi: 10.13366/j.dik.2021.06.021

[31] Corporation S. Zhejiang Launches a major Application Called "Zheli Nursing", Allowing you to make Online Appointments for home care. Available online at: https://cj.sina.com.cn/articles/view/1784473157/6a5ce64502002n5dy (2022).

[32] Tang Z Li W Du C Liu H Wang Y Ma L. Construction and practice of information platform for internet+ NCDs continuous health management mode. China Digit Med. (2023) 18:20–6. doi: 10.3969/j.issn.1673-7571.2023.03.005

[33] Liu T Guo J Liu H Zhang Y Song Y Han X. Construction of the mini program for home care management of chronic wound based on the needs of wound therapists. Chin Nurs Res. (2023) 37:1152–7. doi: 10.12102/j.issn.1009-6493.2023.07.005

[34] Mekbib DB Debeli DK Zhang L Fang S Shao Y Yang W . A novel fully immersive virtual reality environment for upper extremity rehabilitation in patients with stroke. Ann N Y Acad Sci. (2021) 1493:75–89. doi: 10.1111/nyas.14554, 33442915

[35] Tang X. Study on the application effect of PDA system terminal in children intensive care unit of a hospital from 2019 to 2020. J Rare Uncom Dis. (2021) 28:108. doi: 10.3969/j.issn.1009-3257.2021.04.043

[36] Xie L Cheng J Liu X Wu J Zhou T Li Z . Development and application of VR emergency trauma nursing training system. J Nurs Sci. (2024) 39:85–8. doi: 10.3870/j.issn.1001-4152.2024.08.085

[37] Wenqiang Y Fuchao R Guohong S Yuanjing X Tongyou L Youzhuan X . Methods and application of gait analysis of lower limbs after stroke. Chin J Tissue Eng Res. (2023) 27:1257–63. doi: 10.12307/2023.088

[38] Byrne MD. Generative artificial intelligence and ChatGPT. J Perianesth Nurs. (2023) 38:519–22. doi: 10.1016/j.jopan.2023.04.001, 37086240

[39] Tianwei Y Liwen Z Chensu Z Jianrong Y Meihua R. Analysis of technological Progress and industrial development in digital therapeutics. Sci Focus. (2023) 18:14–26. doi: 10.15978/j.cnki.1673-5668.202301005

[40] Administration USFD. Policy for Device Software Functions and Mobile Medical Applications Guidance for Industry and Food and Drug Administration Staff. Available online at: https://www.fda.gov/regulatory-information/search-fda-guidance-documents/policy-device-software-functions-and-mobile-medical-applications (2022).

[41] Zihan W Haoran L Yangguang Z Yishan T Man L. Analysis of the current development status for the digital therapeutics industry. Digit Med Health. (2024) 2:278–83. doi: 10.3760/cma.j.cn101909-20240115-00008

[42] Wang X Li S Zheng X. Application of Mobile APP+blood glucose management model in diabetic patients. Clin Med Eng. (2023) 30:843–4. doi: 10.3969/j.issn.1674-4659.2023.06.0843

[43] Chen H Hong-Ying P. The position-setting and clinical practice of informatics nurse. Chin J Nurs. (2017) 52:546–8. doi: 10.3761/j.issn.0254-1769.2017.05.007

